# Reproducibility and agreement of pulse wave velocity and augmentation index over repeated assessments using two different devices in adolescents

**DOI:** 10.1111/cpf.70049

**Published:** 2026-01-29

**Authors:** Wesley Torres, Earric Lee, Emilia Laitinen, Alan R. Barker, Petri Jalanko, Timo Nurmi, Bert Bond, Ying Gao, Rômulo A. Fernandes, Eero A. Haapala

**Affiliations:** ^1^ Laboratory of Investigation in Exercise (LIVE), São Paulo State University (UNESP) School of Technology and Sciences Presidente Prudente Brazil; ^2^ Sports & Exercise Medicine, Faculty of Sport and Health Sciences University of Jyväskylä Jyväskylä Finland; ^3^ School of Kinesiology and Physical Activity Sciences University of Montréal Montréal Quebec Canada; ^4^ Montreal Heart Institute Montréal Quebec Canada; ^5^ Children's Health and Exercise Research Centre, Public Health and Sport Sciences University of Exeter England UK; ^6^ Helsinki Sports and Exercise Medicine Clinic (HULA) Foundation for Sports and Exercise Medicine Helsinki Finland; ^7^ The Vascular Research Group, Public Health and Sport Sciences University of Exeter England UK; ^8^ Department of Sports Science, College of Education Zhejiang University Hangzhou China; ^9^ Institute of Biomedicine, School of Medicine University of Eastern Finland Kuopio Finland

**Keywords:** cardiovascular health, device agreement, reliability, vascular stiffness, youth

## Abstract

**Objectives:**

We investigated the agreement between pulse wave velocity (PWV) and augmentation index (AIx%) obtained in a controlled fasted condition versus a non‐fasted, uncontrolled, “real‐world” condition. Thereafter, we assessed the reproducibility of PWV and AIx% over three repeated visits under controlled fasted conditions.

**Methods:**

PWV and AIx% were assessed in one uncontrolled visit, and three controlled fasted visits after a 10‐min supine rest in 28 adolescents (61% girls) aged 12–14 years.

**Results:**

Intraclass correlation coefficients (ICCs) between controlled visits were 0.5–0.6 (PWV) and −0.3–0.6 (AIx%) for PulsePen, and 0.5–0.6 (PWV) and 0.4–0.7 (AIx%) for Arteriograph. ICCs between a single uncontrolled visit and controlled visits were lower, especially for the Arteriograph (PWV: 0.0–0.1; AIx%: −0.4–0.0). Device agreement was poor (PWV ICC: −0.2–0.1; AIx% ICC: −0.5–0.3). Reproducibility using PulsePen was (PWV: 0.2–0.8; AIx%: −0.3–0.6) and Arteriograph (PWV: 0.0–0.1; AIx%: −0.4–0.0).

**Conclusion:**

The PulsePen had better reproducibility of PWV than the Arteriograph. Both devices had limited reliability and high variability in AIx% measurements. PWV from the PulsePen may be applicable in healthcare, while results from the Arteriograph should be interpreted with caution. Because of the poor agreement between the devices, the data from these devices should not be directly compared. These results may not be generalizable based on the modest sample size. In addition, the variability in real‐world situations should not be overlooked.

**Abbreviations**
95%CI: 95% confidence intervals
95%LA: 95% limit of agreement
AIx%: augmentation index
BMI: body mass index
CR: coefficient repeatability
CV: coefficient variation
CVD: cardiovascular diseases
DBP: diastolic blood pressure
HR: heart rate
ICC: intraclass correlation coefficients
m/s: metres per second.
M1: measure 1
M2: measure 2
M3: measure 3
PHV: peak height velocity
PWV: pulse wave velocity
SBP: systolic blood pressure
SD: standard deviation
VERNA: Vascular and Brain Health, Exercise, and Nutrition in Adolescents

## 1 INTRODUCTION

Cardiovascular diseases (CVDs) are the leading causes of premature mortality and morbidity (Roth et al., 30), with early signs often emerging as increased arterial stiffness (Hodes et al., 12; Kim and Kim, 16). Early detection of arterial properties in adolescents is crucial for identifying those at elevated cardiovascular risk (Shah et al., 32), as it is useful for detecting arterial stiffness and later cardiac changes. Accurate assessment of arterial properties during adolescence enables timely interventions to improve long‐term cardiovascular health (Haapala et al., 10; Townsend et al., 36). Thus, reliable evaluation of arterial stiffness in youths is vital for proactive prevention strategies (Díaz et al., 6).

Arterial stiffness can be measured non‐invasively by estimating pulse wave velocity (PWV) and augmentation index (AIx%) (Chow and Rabkin, 5; Evangelatos et al., 8). PWV is the velocity of a pressure wave travelling through the artery during each cardiac cycle. Higher PWV is indicative of increased arterial stiffness, as the pulse wave would travel at lower speeds in more distensible arteries (Van Bortel et al., 39). AIx% represents the impact of the reflected wave on systolic blood pressure (Karadağ, 15; Nichols, 25). Increased peripheral vasoconstriction and increased arterial stiffness elevate AIx% because increased peripheral vasoconstriction increases the reflected pressure wave, and stiffer arteries lead to faster reflected pressure wave velocity towards the heart (Buus et al., 3; Casey et al., 4). A higher AIx% increases left ventricular workload as it needs to exert greater pressure to counteract the increased aortic pressure during systole (Pahlevan and Gharib, 27; Sharman et al., 33). Persistently elevated cardiac workload can result in chronic cardiac stress, which over time may quicken the progression of heart failure and left ventricular hypertrophy (Tartière et al., 35).

Carotid‐femoral PWV (cfPWV) is recognized as the most widely accepted measure of arterial stiffness, and can be measured using tonometry‐, mechanotransducer‐, or oscillometry‐based techniques (Townsend et al., 36). The PulsePen is a portable tonometry‐based device used to assess arterial cfPWV in adults (Lee et al., 18; Townsend et al., 36). While the portable nature of the PulsePen device makes it widely accessible, the method requires a highly skilled operator (Pilz et al., 28). In contrast, oscillometry‐based devices that are relatively operator‐free have been developed to mitigate this potential difficulty (Baulmann et al., 1; Nemcsik et al., 24). The Arteriograph is an automatic oscillometric device based on the single‐site brachial cuff analyses used to quantify PWV, AIx%, and central systolic blood pressure (SBPao) (Boutouyrie et al., 2). However, it is important to note that single‐site assessment has been criticized because PWV cannot be directly measured using just one location in the arterial tree, as the pulse wave travel time between two measurement locations is estimated using mathematical algorithms based on age, sex, stature, and blood pressure (Jin et al., 14).

PWV and AIx% have been reasonably well‐established as reproducible measures of arterial stiffness and CVD risk assessment in adults (Horváth et al., 13; Niwińska and Chlabicz, 26). However, a significant research gap in between‐device agreement and reproducibility for paediatric populations still exists (Townsend et al., 36; Urbina et al., 38). An earlier study reported acceptable reproducibility of PWV and AIx% over two visits within 2 weeks among adolescents using interclass correlation coefficients (ICCs) above 0.75 (Koo and Li, 17; Lowenthal et al., 19). Our prior findings also show good short‐term reproducibility for aortic PWV and moderate reproducibility for AIx% in adolescents aged 16–19 years during a single laboratory visit (Haapala et al., 10). Nevertheless, the reproducibility of more than two repeated measures remains unknown. Understanding how these measures hold up across different visits will help to determine whether a measurement method produces consistent results over time and under various real‐world conditions. Moreover, most studies have utilized fasted and highly standardized conditions for their assessments, and it is unclear if the results from different devices are similar between fasted controlled and non‐fasted, ‘real‐world,’ uncontrolled conditions. From a practical standpoint, investigating and understanding the effects of participant conditions would improve the practical applicability of these methods, for example, in school healthcare (Haapala et al., 10; Horváth et al., 13). Laboratory environments better discriminate vascular alterations, supporting early CVD risk detection, but may underestimate real‐life stiffness influenced by activity (Townsend et al., 2015). Real‐world PWV tracking via wearables promises longitudinal monitoring of daily behaviours yet requires corrections for posture/activity to match lab gold standards (Marshall et al., 20).

Understanding the reproducibility and agreement of arterial stiffness analysis data from different devices can also enhance the reporting of arterial stiffness in research, provide information on the strengths and limitations of the current methods, improve the identification of adolescents at increased CVD risk, and translate the research to viable guidelines for clinical practice. Therefore, we: (1) explored the reproducibility of PWV and AIx% between the fasted controlled and non‐fasted uncontrolled conditions using the Arteriograph and the PulsePen devices. (2) investigated the reproducibility of PWV and AIx% over three controlled visits in fasted conditions, and finally, we also (3) compared the agreement of PWV and AIx% between the Arteriograph and the PulsePen devices.

## 2 METHODS

### 2.1 Study population and design

The present analyses were based on the data from the Vascular and Brain Health, Exercise, and Nutrition in Adolescents (VERNA) study (ISRCTN11803379). Altogether 28 adolescents (61% girls) aged 12–14 from the City of Jyväskylä, Finland, participated in the baseline examinations, which took place in the exercise and health laboratory at the Faculty of Sport and Health Sciences of the University of Jyväskylä. The baseline examinations included the assessments of PWV and AIx%, body size and composition, and maximal oxygen uptake (VO_2max_). The Research Ethics Committee of the Hospital District of Central Finland, Jyväskylä, approved the study protocol in 2022 (5U/2021). The parents and/or caregivers of the adolescents gave their written informed consent, and the adolescents gave their assent for participation. The VERNA study was carried out in accordance with the principles of the Declaration of Helsinki, as revised in 2008. The inclusion criteria were: (1) aged 12–14 years and (2) apparently healthy. The health status was confirmed using a standardized physical activity readiness questionnaire.

### 2.2 Protocol

**Figure 1:**
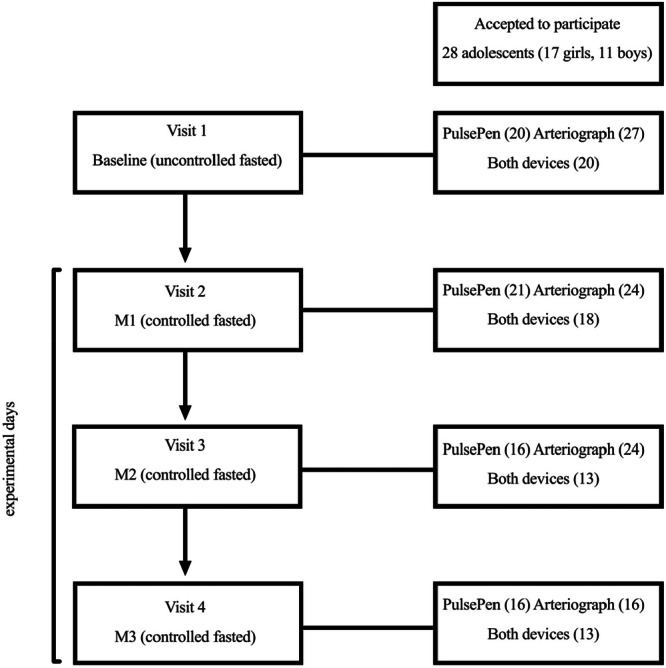
Flowchart of pulse wave velocity and augmentation index measurements with the PulsePen and Arteriograph devices. M1, measure 1; M2, measure 2; M3, measure 3.

For the baseline examinations (i.e., non‐fasted uncontrolled condition), the adolescents were instructed to fast for 2–3 h before the assessments and to avoid moderate‐to‐vigorous exercise that may affect the VO_2max_ testing. The baseline examinations took place between 09:00 and 19:00, depending on the adolescents' schedules. The initial measurement period was under non‐fasting, non‐standardized conditions. The single assessment time point was conducted during the ‘uncontrolled fasted’ condition at baseline. The subsequent period where measurements were taken under standardized, fasting conditions according to the study protocol. The sequential assessment time points were conducted during the ‘controlled fasted’ condition at measurements 1 to 3 (M1–M3) at the Magnetoencephalography laboratory at the Centre for Interdisciplinary Brain Research, University of Jyväskylä, with a minimum of 2 days between each visit (Figure 1). The adolescents were instructed to avoid moderate‐to‐vigorous physical activity and exercise a day before the visits and to maintain their normal dietary habits. Adolescents arrived with family transportation at the laboratory between 08:00 and 09:00 after an overnight fast. Upon arrival, they were asked to rest in a supine position for 10 min before the assessment of PWV and AIx%. After the first measurement, the adolescents had a standardized breakfast. In the present study, we used only the first fasted controlled state measurement from all the visits in the analyses.

The main analysis focused on the relationship in question of the reproducibility of PWV and AIx%, so we calculated the sample size using expected ICCs for PWV reliability from previous (Haapala et al., 10). Assuming the lowest ICC observed in the study (ICC = 0.75), alpha of 0.05, and 80% of statistical power (Miot, 22; Walter et al., 41), the minimal sample size required was 12 participants.

### 2.3 Assessment of PWV and augmentation index

PWV and AIx% were assessed using the non‐invasive oscillometric ArterioGraph (TensioMed Ltd.) and arterial tonometer PulsePen (DiaTecne s.r.l., Milan, Italy) devices. The software used was TensioMed Arteriograph (version 3.0.1.1). The Arteriograph and PulsePen were validated by comparing their PWV measurements with those from invasive catheterization, which offers the most precision and accuracy (Horváth et al., 13; Salvi et al., 31).

Firstly, an oscillometric pulse wave analysis was performed on the left and right upper arms. The Arteriograph device provides an automatic assessment of resting heart rate (HR), systolic blood pressure (SBP), diastolic blood pressure, mean arterial pressure, pulse pressure, aortic pulse wave velocity (PWVao), and AIx%. The device begins by measuring SBP, followed by suprasystolic occlusion, where the cuff is inflated to 35 mmHg above the measured SBP. Thereafter, pulse waves from the brachial artery are detected via a piezoelectric sensor. The signals are then passed on to a laptop computer, recorded, and analyzed. The computer software calculates PWVao (m/s) using the time difference between the first systolic wave (direct) and the second systolic wave (reflected) in relationship to the distance from the jugulum to the pubic symphysis. AIx% was computed from the pressure difference between the first (P1) and second (P2) wave in relation to the pulse pressure by the formula AIx% = [(P2 − P1)/pulse pressure] × 100. All oscillometric measurements were taken by trained researchers (E. Lee and E. Laitinen).

Secondly, a tonometric carotid‐femoral pulse wave analysis was performed immediately after oscillometric measurement. The Pulse Pen device is composed of one tonometer and an integrated ECG unit. The PulsePen is made of a pressure probe the size and shape of a ballpoint pen with a built‐in acquisition device that serves to non‐invasively detect the pressure waveform by means of applanation tonometry. PWV was measured by recording carotid and femoral waveforms in rapid succession at a sample rate of 1 kHz, and defined as the transit distance between the measuring sites divided by the time delay between the distal pulse and proximal pulse wave, using the ECG trace as reference. Transit distances were assessed by measuring the distance from the suprasternal notch to each pulse recording site (carotid and femoral) using a tape measure. Direct carotid to femoral measurement was adjusted to 80% (common carotid artery–common femoral artery×0.8) for the calculation of PWV as recommended by current guidelines (Spronck et al., 34). Transit time was defined as the difference between the delay of the distal and proximal pulse wave, both belonging to the ECG qRs complex. The pulse wave delay was determined by calculating the time elapsed from the peak of the R wave and the ‘foot’ of the pressure pulse wave. All tonometry measurements were taken by a single operator (E. Lee) of the tonometer to minimize ascertainment biases and to ensure consistency and reliability.

### 2.4 Assessment of body size, body composition, and somatic maturity

Body mass, lean body mass, fat mass, and body fat percentage were measured with the adolescent having fasted for at least 3 h, emptied the bladder, and standing in light underwear using a calibrated InBody® 770 bioelectrical impedance device (Biospace) to an accuracy of 0.1 kg. Stature was measured with the participant standing in the Frankfurt plane without shoes using a wall‐mounted stadiometer to an accuracy of 0.1 cm. Body mass index was calculated by dividing weight (kg) by height (m) squared. Somatic maturity status in terms of time to peak height velocity was calculated using the equations by Moore et al., 23.

### 2.5 Statistical analyses

Descriptive statistics, including mean values and 95% confidence intervals (95% CI), were calculated for the participants' characteristics. We studied reproducibility using

- intraclass correlation coefficients (ICC) and their 95% CI to quantify reliability by assessing agreement between multiple measures,
- coefficient variation with the root‐mean‐square method expressed as percentages (CV%) to measure variability in repeated measures,
- Wilcoxon test and 95% limit of agreement (95% LA) to detect systematic differences,
- the mean absolute percent errors and 95% CI to evaluate forecast accuracy as relative error,
- coefficient repeatability (CR) to measure repeatability and
- line of equality with 95% CI of the Bland–Altman plot.

using the MedCalc Statistical Software version 17.8.6 (MedCalc Software bvba, Ostend, Belgium). Measures of interpreting agreement of ICCs values were as follows: below 0.50 (poor), between 0.50 and 0.75 (moderate), between 0.75 and 0.90 (good), and above 0.90 (excellent) (Koo and Li, 17). We also investigated whether the agreement between the two devices' measurements in PWVao and AIx% was proportionally related to the sample mean using the regression line of the difference in the Bland–Altman plot.

## 3 RESULTS

### 3.1 Participant characteristics

Twenty‐eight adolescents participated in our study. They were, on average, 13.1 (12.9 to 13.4) years old and had a positive peak height velocity (reached maturity offset). The characteristics of the participants are described in Table 1.

**Table 1:** Characteristics of the sample.

	All (*n* = 28 [boys = 11, girls = 17])
	Mean (SD)
*Anthropometric and body composition variables*	
Age (years)	13.1 (0.7)
Stature (cm)	159.5 (9.6)
Body weight (kg)	50.7 (11.9)
PHV	0.5 (0.9)
BMI	19.7 (3.2)
Fat mass (kg)	10.7 (6.7)
Body fat (%)	20.2 (8.5)
Lean body mass (kg)	37.6 (7.9)
*Hemodynamic variables*	Mean (95% CI)
HR (beats/min)	
Baseline	72 (67; 76)
M1	67 (62; 72)
M2	68 (64; 73)
M3	66 (61; 70)
SBP (mmHg)	
Baseline	108 (104; 113)
M1	105 (102; 107)
M2	104 (101; 108)
M3	104 (101; 106)
DBP (mmHg)	
Baseline	59 (56; 62)
M1	54 (52; 56)
M2	57 (55; 59)
M3	55 (53; 57)
Pulse wave velocity (m/s)	
*PulsePen*	
Baseline (*n* = 20 [boys = 7, girls = 13])	5.55 (5.26; 5.84)
M1 (*n* = 21 [boys = 10, girls = 11])	5.53 (5.18; 5.87)
M2 (*n* = 16 [boys = 6, girls = 10])	5.49 (5.07; 5.90)
M3 (*n* = 16 [boys = 6, girls = 10])	5.55 (5.09; 6.00)
*Arteriograph*	
Baseline (*n* = 27 [boys = 10, girls = 17])	6.15 (5.82; 6.48)
M1 (*n* = 24 [boys = 11, girls = 13])	5.70 (5.53; 5.88)
M2 (*n* = 24 [boys = 11, girls = 23])	5.66 (5.41; 5.90)
M3 (*n* = 22 [boys = 11, girls = 11])	5.57 (5.37; 5.77)
Augmentation index (%)	
*PulsePen*	
Baseline (*n* = 17 [boys = 7, girls = 10])	14.33 (8.87; 19.78)
M1 (*n* = 20 [boys = 10, girls = 10])	14.04 (10.21; 17.87)
M2 (*n* = 17 [boys = 6, girls = 11])	10.87 (7.47; 14.27)
M3 (*n* = 16 [boys = 6, girls = 10])	15.28 (11.85; 18.70)
*Arteriograph*	
Baseline (*n* = 26 [boys = 9, girls = 17])	10.55 (8.19; 12.91)
M1 (*n* = 24 [boys = 11, girls = 13])	10.11 (7.68; 12.53)
M2 (*n* = 24 [boys= 11, girls = 13])	9.52 (7.51; 11.53)
M3 (*n* = 22 [boys = 11, girls = 11])	9.38 (6.59; 12.17)

Abbreviations: BMI, body mass index; DBP, diastolic blood pressure; HR, resting heart rate; M1, measure 1; M2, measure 2; M3, measure 3; m/s, metres per second; PHV, peak height velocity; SBP, systolic blood pressure; SD, standard deviation; 95%CI, 95% confidence interval.

### 3.2 Reproducibility of PWV and AIx% between the controlled fasted visits

**Figure 2:**
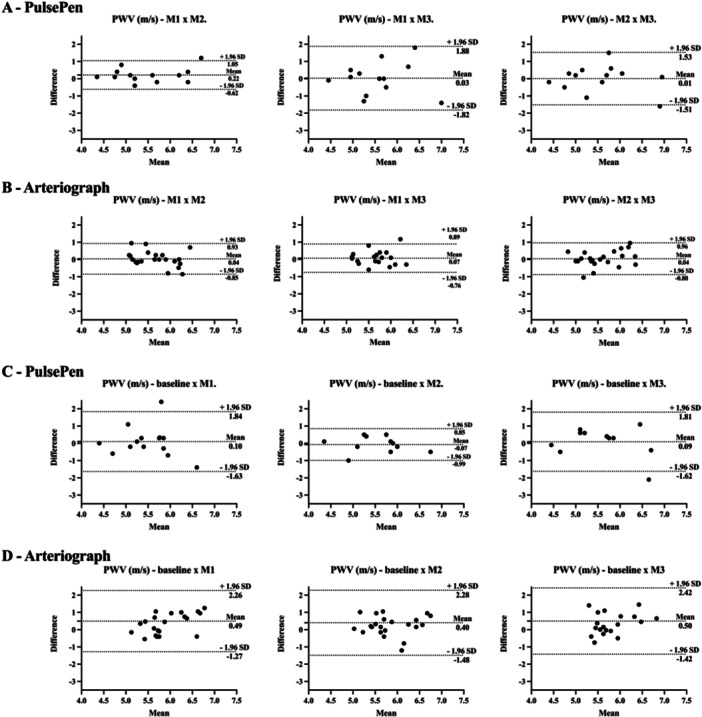
Bland–Altman plots of pulse wave velocity by the PulsePen and Arteriograph devices with the mean difference (95% confidence intervals) and limits of agreement (95% confidence intervals). PWV: pulse wave velocity; m/s = metres per second; M1 = measure 1; M2 = measure 2; M3 = measure 3. A: measurements under controlled fasted conditions using PulsePen device; B: measurements under controlled fasted conditions using Arteriograph device; C: measurements under uncontrolled and controlled fasted conditions using PulsePen device; D: measurements under uncontrolled and controlled fasted conditions using Arteriograph device.

**Figure 3:**
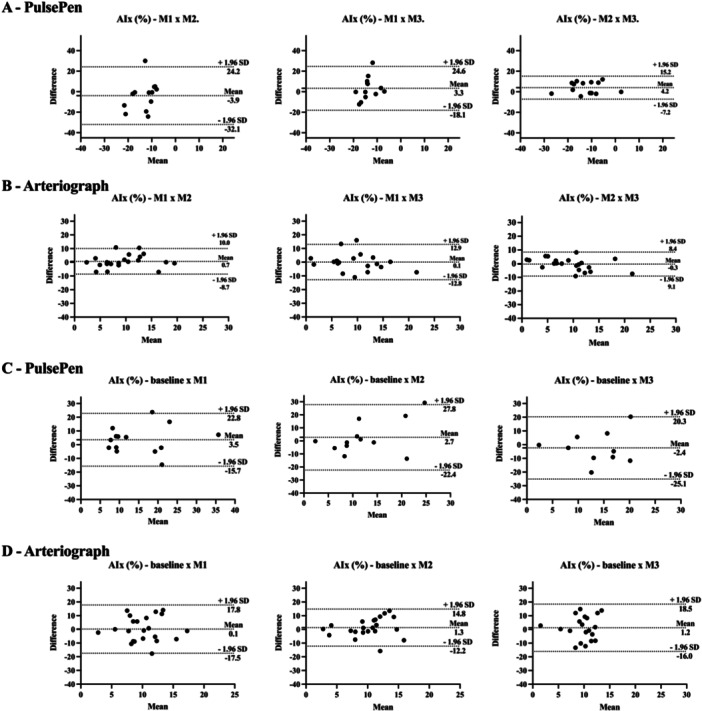
Bland–Altman plots of augmentation index by the PulsePen and Arteriograph devices with the mean difference (95% confidence intervals) and limits of agreement (95% confidence intervals). AIx%: augmentation index; m/s = metres per second; M1 = measure 1; M2 = measure 2; M3 = measure 3. A: measurements under controlled fasted conditions using PulsePen device; B: measurements under controlled fasted conditions using Arteriograph device; C: measurements under uncontrolled and controlled fasted conditions using PulsePen device; D: measurements under uncontrolled and controlled fasted conditions using Arteriograph device.

For the PulsePen device, ICCs for PWV between the controlled fasting visits varied from 0.3 (poor) to 0.8 (good) and CV% from 5% to 11% (Table 2). The AIx% corresponding values were −0.3 (poor) to 0.6 (moderate), and 50% to 70%, respectively (Table 3). No statistically significant changes in PWV or AIx% were observed between controlled fasting measurements in the Wilcoxon test (Figure 2 and Figure 3).

**Table 2:** Reproducibility and comparison of pulse wave velocity using the PulsePen and Arteriograph devices.

	ICC (95%CI)	CV% (95%CI)	95% LA	CR (95%CI)	*p*‐Value from Wilcoxon test
*PulsePen*					
Baseline – M1 (*n* = 14)	0.25 (−0.33 to 0.68)	10.5 (0.0 to 15.6)	−1.63 and 1.84	1.68 (1.23 to 2.65)	0.779
Baseline – M2 (*n* = 11)	0.78 (0.37 to 0.93)	6.0 (0.0 to 8.7)	−0.99 and 0.85	0.88 (0.62 to 1.50)	0.643
Baseline – M3 (*n* = 11)	0.53 (−0.08 to 0.85)	9.7 (0.0 to 13.8)	−1.62 and 1.81	1.64 (1.16 to 2.79)	0.349
M1 – M2 (*n* = 13)	0.82 (0.51 to 0.94)	5.6 (0.2 to 8.0)	−0.62 and 1.05	0.90 (0.65 to 1.46)	0.113
M1 – M3 (*n* = 13)	0.37 (−0.23 to 0.76)	10.8 (5.9 to 14.1)	−1.81 and 1.87	1.77 (1.28 to 2.86)	0.893
M2 – M3 (*n* = 13)	0.61 (0.10 to 0.86)	9.0 (3.3 to 12.3)	−1.51 and 1.53	1.46 (1.05 to 2.35)	0.673
*Arteriograph*					
Baseline – M1 (*n* = 23)	0.13 (−0.18 to 0.47)	10.8 (0.0 to 15.4)	−1.27 and 2.26	1.98 (1.54 to 2.94)	**0.010**
Baseline – M2 (*n* = 23)	0.10 (−0.24 to 0.46)	11.1 (0.0 to 16.4)	−1.48 and 2.28	2.00 (1.55 to 2.80)	**0.020**
Baseline – M3 (*n* = 21)	0.00 (−0.32 to 0.37)	11.8 (0.0 to 17.1)	−1.42 and 2.42	2.11 (1.62 to 3.02)	**0.019**
M1 – M2 (*n* = 22)	0.59 (0.23 to 0.81)	5.5 (3.0 to 7.2)	−0.85 and 0.93	0.87 (0.67 to 1.23)	0.585
M1 – M3 (*n* = 20)	0.52 (0.11 to 0.77)	5.0 (2.2 to 6.8)	−0.76 and 0.89	0.81 (0.62 to 1.17)	0.708
M2 – M3 (*n* = 22)	0.60 (0.24 to 0.81)	5.8 (3.2 to 7.5)	−0.88 and 0.96	0.90 (0.69 to 1.27)	0.638
*Arteriograph and PulsePen devices*					
Baseline (*n* = 20)	0.10 (−0.15 to 0.42)	14.1 (7.9 to 18.2)	−2.69 and 1.02	2.44 (1.87 to 3.53)	**0.001**
M1 (*n* = 18)	−0.11 (−0.55 to 0.36)	11.6 (8.2 to 14.2)	−1.95 and 1.68	1.78 (1.35 to 2.60)	0.468
M2 (*n* = 13)	0.16 (−0.35 to 0.61)	11.8 (4.3 to 16.2)	−2.02 and 1.57	1.78 (1.32 to 2.76)	0.468
M3 (*n* = 13)	−0.19 (−0.70 to 0.38)	12.6 (7.4 to 16.2)	−2.00 and 2.20	2.02 (1.48 to 3.19)	0.850

*Note*: *p*‐values were calculated using the Wilcoxon signed‐rank test.

Statistically significant values (*p* < 0.05) are given in bold.

Abbreviations: CR, coefficient of repeatability; CV%, coefficient variation; ICC, intraclass correlation coefficient; M1, measure 1; M2, measure 2; M3, measure 3; m/s, metres per second; 95% CI, 95% confidence interval; 95% LA, 95% limits of agreement.

**Table 3:** Reproducibility and comparison of augmentation index using the PulsePen and Arteriograph devices.

	ICC (95%CI)	CV% (95%CI)	95% LA	CR (95%CI)	*p*‐value from Wilcoxon test
*PulsePen*					
Baseline – M1	0.46 (−0.02 to 0.78)	48.4 (22.5 to 64.6)	−15.7 and 22.8	19.78 (14.48 to 31.21)	0.140
Baseline – M2	0.04 (−0.55 to 0.58)	57.9 (27.7 to 77.0)	−22.4 and 27.8	24.61 (17.64 to 40.62)	0.909
Baseline – M3	−0.03 (−0.68 to 0.59)	52.8 (4.5 to 74.5)	−25.1 and 20.3	22.06 (15.41 to 38.71)	0.431
M1 – M2	−0.32 (−0.74 to 0.25)	70.1 (26.7 to 95.5)	−22.2 and 30.8	26.79 (19.42 to 43.16)	0.339
M1 – M3	−0.36 (−0.77 to 0.22)	49.9 (11.2 to 69.6)	−22.7 and 16.8	19.84 (14.38 to 31.96)	0.497
M2 – M3	0.58 (0.08 to 0.84)	55.4 (0.0 to 82.4)	−15.2 and 7.2	13.33 (9.76 to 21.03)	0.058
*Arteriograph*					
Baseline – M1	−0.33 (−0.69 to 0.11)	63.0 (45.9 to 76.3)	−17.5 and 17.8	17.21 (13.31 to 24.35)	0.986
Baseline – M2	0.00 (−0.41 to 0.42)	44.9 (31.0 to 55.4)	−12.2 and 14.8	13.45 (10.40 to 19.04)	0.417
Baseline – M3	−0.46 (−0.78 to −0.01)	67.5 (47.2 to 82.9)	−16.0 and 18.5	16.97 (12.98 to 24.51)	0.570
M1 – M2	0.57 (0.21 to 0.80)	42.8 (16.7 to 58.2)	−8.7 and 10.0	9.23 (7.13 to 13.06)	0.780
M1 – M3	0.40 (‐0.04 to 0.71)	70.4 (2.9 to 102.4)	−12.8 and 12.9	12.55 (9.60 to 18.13)	0.851
M2 – M3	0.67 (0.35 to 0.84)	64.0 (0.0 to 92.8)	−9.1 and 8.4	8.60 (6.65 to 12.17)	0.848
*Arteriograph and PulsePen devices*					
Baseline	−0.54 (−0.85 to −0.04)	79.3 (59.8 to 94.9)	−34.3 and 27.4	30.63 (22.81 to 46.62)	0.632
M1	0.07 (−0.29 to 0.47)	56.9 (30.8 to 70.5)	−13.2 and 23.2	20.23 (15.18 to 30.33)	**0.003**
M2	0.34 (−0.23 to 0.73)	63.9 (0.0 to 90.4)	−13.3 and 14.3	13.33 (9.76 to 21.02)	0.541
M3	−0.03 (−0.51 to 0.49)	60.2 (0.0 to 85.7)	−13.7 and 19.9	17.25 (12.51 to 27.80)	0.216

*Note*: *p*‐values were calculated using the Wilcoxon signed‐rank test.

Statistically significant values (*p* < 0.05) are given in bold.

Abbreviations: CR, coefficient of repeatability; CV%, coefficient variation; ICC, intraclass correlation coefficient; M1, measure 1; M2, measure 2; M3, measure 3; 95% CI, 95% confidence interval; 95% LA, 95% limits of agreement.

For the Arteriograph, the ICCs for PWV ranged from 0.5 (moderate) to 0.6 (moderate) and CV% from 5.0% to 5.8% (Table 2). The AIx% corresponding values were 0.4 (poor) to 0.7 (moderate) and 43% to 64%, respectively (Table 3). No statistically significant changes in PWV or AIx% were observed between controlled fasting measurements in the Wilcoxon test (Figure 2 and Figure 3).

### 3.3 Reproducibility of PWV and AIx% between the uncontrolled fasted and the controlled fasted conditions

For the PulsePen device, ICCs for PWV between the uncontrolled non‐fasting baseline and controlled fasting visits ranged from 0.2 (poor) to 0.8 (good), and CV% from 6% to 11% (Table 2). The AIx% corresponding values were −0.3 (poor) to 0.6 (moderate) and 48% to 58%, respectively (Table 3). No statistically significant changes in PWV or AIx% were observed between uncontrolled and controlled fasting measurements in the Wilcoxon test (Figure 2 and Figure 3).

For the Arteriograph, the ICCs for PWV ranged from 0.0 (poor) to 0.1 (poor) and CV% from 10% to 12% (Table 2). The AIx% corresponding values were −0.4 (poor) to 0.0 (poor) and 44.9% to 67.4%, respectively (Table 3). Baseline PWV was different from that assessed at M1, M2, and M3 (*p* = 0.010, 0.020, and 0.019, respectively). No statistically significant changes in PWV or AIx% were observed between uncontrolled and controlled fasting measurements in the Wilcoxon test (Figure 2 and Figure 3).

### 3.4 Agreement of PWV and AIx% between the devices

**Figure 4:**
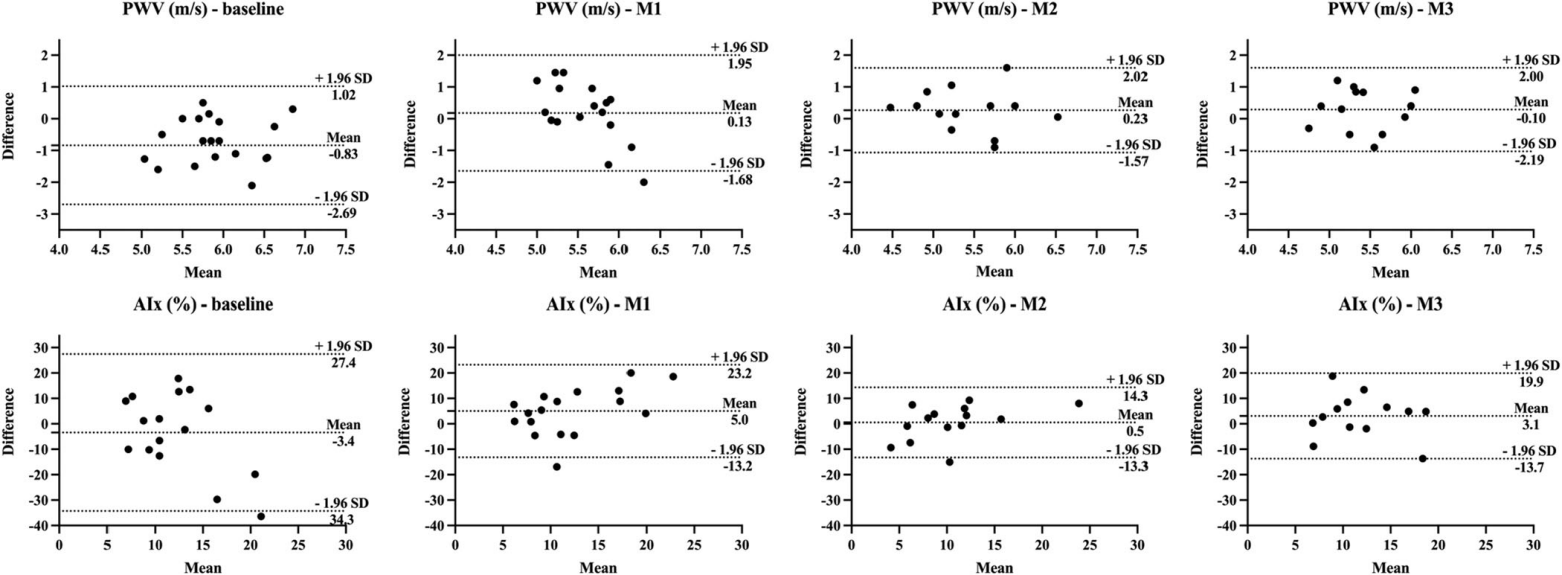
Bland–Altman plots of pulse wave velocity and augmentation index between Arteriograph and PulsePen devices with the mean difference (95% confidence intervals) and limits of agreement (95% confidence intervals). AIx%, augmentation index; m/s, metres per second; M1, measure 1; M2, measure 2; M3, measure 3; PWV, pulse wave velocity.

The ICCs for PWV between devices ranged from −0.2 (poor) to 0.1 (poor), and CV% from 11% to 14% (Table 2). The AIx% corresponding values range from −0.5 (moderate) to 0.3 (poor) and 57% to 79%, respectively (Table 3). The Wilcoxon test suggests a significant difference between devices for PWV in the baseline measurement and AIx% during M1 (Figure 4).

## 4 DISCUSSION

We found that the PWV across visits in the fasted controlled state, the ICCs ranged between 0.3 (poor) to 0.8 (good), and CV% 5.6 to 10.8 for the PulsePen device; the ICCs ranged between 0.5 (moderate) to 0.6 (moderate), and CV% 5.0 to 5.8 for the Arteriograph device. However, the reproducibility of AIx% in both devices was much poorer than that of PWV. We also observed that the reproducibility between fasted controlled and non‐fasted uncontrolled measurements varied between the devices, with better reproducibility for the PulsePen device than for the Arteriograph device. The wide CIs surrounding our ICCs have critical implications for interpreting the reliability and reproducibility of our findings. Finally, our findings suggest that the devices are not comparable for PWV and AIx% measurements.

During the controlled fasted condition (M1 to M3), the PulsePen demonstrated greater agreement in PWV values between M1 and M2 (ICC = 0.82), but the reproducibility between other visits was poorer, ICCs ranging from 0.3 to 0.6, with CV% ranging between 5.6% to 10.8%. We found that the Arteriograph had low to moderate reproducibility from ICCs 0.5 to 0.6 in PWV assessment, with CV% ranging between 5.0% to 5.7%. Moreover, both devices had high variability and poor reproducibility for AIx%. Nevertheless, our findings agree relatively well with the results of studies comparing the reproducibility between 2 days, either using the arterial applanation tonometry, in which the ICCs were 0.61 for PWV and 0.78 for AIx% (Lowenthal et al., 19), or using the finger pulse wave analysis, where the CV% for stiffness index was 6.3 (Veijalainen et al., 40). Overall, PWV in the PulsePen device had moderate to good reproducibility, while the Arteriograph device showed low to moderate reproducibility, despite the standardization of the measurement conditions. This suggests that the between‐visit comparisons of the Arteriograph device should be interpreted with caution.

When we compared the agreement between PWV and AIx% between the uncontrolled fasted and the controlled fasted conditions, the PulsePen device showed moderate to good reproducibility in PWV measurements, but AIx% measurements had greater variability and were less reliable. Nevertheless, the analysis did not detect a statistically significant difference between the baseline day and fasted controlled visits, while this result is compatible with the device's consistency over time. However, the variability in AIx% does affect its overall reliability. Therefore, our results agree with the previous studies in adults that PWV assessed by the PulsePen device may be reliable to use in clinical practice also in uncontrolled environments (Salvi et al., 31). However, it must be noted that AIx% values derived from the device may have limited reproducibility.

The Arteriograph showed poor reproducibility in both PWV and AIx% measurements between the uncontrolled fasted and the controlled fasted conditions, with high variability and low ICC values, suggesting that the device may not be reliable for repeated assessments in uncontrolled conditions. This variability can result from changes in physiological status, such as heart rate and blood pressure, and reliance on the algorithm estimating pulse wave travel time based on age, sex, stature, and blood pressure (Milan et al., 21). Given that arteries are dynamic structures that respond to changes in shear stress, conditions in which the blood flow is significantly altered (i.e. after exercise or post‐prandial) would lead to even greater discrepancies (Elliot et al., 7). These limitations should be acknowledged when using Arteriograph in clinical and health care settings (Milan et al., 21; Podrug et al., 29).

We found a poor agreement in PWV between the devices. Moreover, AIx% values showed inferior agreement between the devices. Devices that use technology and algorithms based on measurements from two sites, such as the PulsePen, may be less affected by changes to the peripheral vascular system (Salvi et al., 31). On the other hand, oscillometric devices estimate PWV by analyzing peripheral pressure waves from a single site; this estimated PWV has a strong dependence on age and systolic BP and uses estimates of pulse wave travel time (Grillo et al., 9; Niwińska and Chlabicz, 26). These different vascular sites impact PWV and AIx values because of the arterial path and the technical approach to waveform analysis and distance estimation. Therefore, our findings suggest that the two devices should not be used interchangeably to assess PWV or AIx%, in line with the ARTERY Society Guidelines (Townsend et al., 36; Wilkinson et al., 42).

The strengths of the study include the assessment of PWV and AIx% between non‐fasted uncontrolled and fasted controlled visits and the comparison between two non‐invasive devices—the validated and widely accepted applanation tonometry method of the PulsePen device and an operator‐free oscillometry‐based assessment of the Arteriograph device. The same skilled operator also performed all the pulse wave analyses of the PulsePen device (E. Lee), which further reduces the operator‐related variability. Some limitations should be recognized, such as an unbalanced sex distribution. In addition, we lacked the sample size to perform sex‐stratified analyses. Missing data from the PulsePen device reduced statistical power and created a potential bias. Moreover, because the PWV values were within the normal range (Hidvégi et al., 11), the results cannot be generalized for populations with increased arterial stiffness. The number of participants with complete data varied across the assessment time points, and the interpretation of our results must be considered in the context of significant participant attrition over the course of the study.

In conclusion, the reproducibility of PWV in the PulsePen device was poor to good, and in the Arteriograph device was moderate in adolescents, according to ICCs. The agreement between the devices was low. Both devices demonstrated limited reliability and high variability in AIx% measurements. Our study was limited by attrition, leading to a varying number of subjects across the different measurement time points. Furthermore, the sex distribution within the analyzed sample was not consistent over time. This imbalance could potentially introduce bias and affect the generalizability of our findings, particularly for the later follow‐up assessments. Finally, PWV assessed using the PulsePen device may be applicable in healthcare settings under uncontrolled and non‐fasted conditions, whereas results obtained from Arteriograph assessments should be interpreted with caution. Therefore, our findings highlight the potential of this method but strongly emphasize the need for further, larger‐scale studies specifically designed to confirm and precisely quantify its reliability before it can be confidently adopted in clinical or research practice. Overall, these findings highlight certain limitations of both devices, indicating that they may not be entirely comparable and should not be used interchangeably.

## CONFLICT OF INTEREST STATEMENT

The authors declare no conflicts of interest.

## Data Availability

The data that support the findings of this study are available from the corresponding author upon reasonable request.
